# Association between hospital case volume and mortality in non-elderly pneumonia patients stratified by severity: a retrospective cohort study

**DOI:** 10.1186/1472-6963-14-302

**Published:** 2014-07-12

**Authors:** Hiraku Kumamaru, Yusuke Tsugawa, Hiromasa Horiguchi, Kanako Kunishima Kumamaru, Hideki Hashimoto, Hideo Yasunaga

**Affiliations:** 1 Department of Health and Social Behavior, School of Public Health, The University of Tokyo, Tokyo, Japan; 2 Harvard Interfaculty Initiative in Health Policy, Cambridge, MA, USA; 3 Department of Clinical Data Management and Research, Clinical Research Center, National Hospital Organization Headquarters, Tokyo, Japan; 4 Applied Imaging Science Laboratory, Department of Radiology, Brigham and Women’s Hospital and Harvard Medical School, Boston, MA, USA; 5 Department of Clinical Epidemiology and Health Economics, School of Public Health, The University of Tokyo, 7-3-1 Hongo, Bunkyo-ku, Tokyo 1138655, Japan

**Keywords:** Age-factors, Hospital volume, Mortality, Non-elderly, Pneumonia, Quality of care, Effect modification

## Abstract

**Background:**

The characteristics and aetiology of pneumonia in the non-elderly population is distinct from that in the elderly population. While a few studies have reported an inverse association between hospital case volume and clinical outcome in elderly pneumonia patients, the evidence is lacking in a younger population. In addition, the relationship between volume and outcome may be different in severe pneumonia cases than in mild cases. In this context, we tested two hypotheses: 1) non-elderly pneumonia patients treated at hospitals with larger case volume have better clinical outcome compared with those treated at lower case volume hospitals; 2) the volume-outcome relationship differs by the severity of the pneumonia.

**Methods:**

We conducted the study using the Japanese Diagnosis Procedure Combination database. Patients aged 18–64 years discharged from the participating hospitals between July to December 2010 were included. The hospitals were categorized into four groups (very-low, low, medium, high) based on volume quartiles. The association between hospital case volume and in-hospital mortality was evaluated using multivariate logistic regression with generalized estimating equations adjusting for pneumonia severity, patient demographics and comorbidity score, and hospital academic status. We further analyzed the relationship by modified A-DROP pneumonia severity score calculated using the four severity indices: dehydration, low oxygen saturation, orientation disturbance, and decreased systolic blood pressure.

**Results:**

We identified 8,293 cases of pneumonia at 896 hospitals across Japan, with 273 in-hospital deaths (3.3%). In the overall population, no significant association between hospital volume and in-hospital mortality was observed. However, when stratified by pneumonia severity score, higher hospital volume was associated with lower in-hospital mortality at the intermediate severity level (modified A-DROP score = 2) (odds ratio (OR) of very low vs. high: 2.70; 95% confidence interval (CI): 1.12–6.55, OR of low vs. high: 2.40; 95% CI:0.99–5.83). No significant association was observed for other severity strata.

**Conclusions:**

Hospital case volume was inversely associated with in-hospital mortality in non-elderly pneumonia patients with intermediate pneumonia severity. Our result suggests room for potential improvement in the quality of care in hospitals with lower volume, to improve treatment outcomes particularly in patients admitted with intermediate pneumonia severity.

## Background

Pneumonia remains a substantial burden in developed countries [1,2], not only in the elderly, but also in younger patients. Pneumonia in the non-elderly population is known to have a different aetiology from that in the elderly population, and life-threatening cases usually occur in the presence of underlying chronic illnesses [3-6]. Early diagnosis followed by early initiation of appropriate antibiotics may improve the outcome in this population, but heterogeneous and non-specific presentations, also a lack of suspicion, often results in misinterpretation of symptoms [5] and delayed treatment. A study from the UK reported management deficiencies in younger patients dying from pneumonia, [3] suggesting there was room for improvement in their care which might lead to improved clinical outcome.

Many previous studies have reported that higher hospital case volume was associated with better outcomes in surgical procedures [7-10], non-surgical interventional procedures [11-14], medical treatments [15,16], and in pneumonia in the elderly population [16-18]. Although the evidence is still scarce on the mechanism of the relationship, larger case volume hospital are thought to be associated with better outcome for a number of reasons, such as better standardized care complying with recommended practice guidelines [17,19,20], increased use of peri-procedural testing, monitoring, or preventive processes [21], and care by physicians with greater clinical experience and skill. We hypothesized that in non-elderly pneumonia patients, there was also an inverse relationship between case volume and outcome, as providers with greater experience would likely be more aware of the risks associated with mismanagement of the treatment in these cases.

The relationship between hospital case volume and clinical outcome can also be modified by disease severity. Compared with patients undergoing elective surgeries with specific indications for the procedure, medically treated patients are admitted to hospital with a much wider variety of severity levels. This leads to the increased importance of severity adjustment for case mix when examining the overall volume-outcome relationship, as well as a potential for modified effect of quality of care on outcome depending on the severity of the disease at admission. A few previous studies evaluated the effect of disease severity on the relationship between process of care measures and outcome for elderly pneumonia patients [22,23] or patients with other medically treated diseases [24], with mixed results. It is possible that a population of non-elderly pneumonia patients might show a differential volume-outcome effect according to pneumonia severity at admission, especially when looking at mortality as an outcome, since mild cases of pneumonia are unlikely to be fatal in this population.

Hence, the purpose of this study was: 1) to evaluate the relationship between hospital case volume and in-hospital mortality of non-elderly adult patients aged 18–64 years, hospitalized for pneumonia in Japan; and 2) to evaluate the volume-outcome relationship in groups stratified by pneumonia severity.

## Methods

### Data source

We utilized the Japanese Diagnosis Procedure Combination (DPC) database for the study. The DPC Database is a nationwide inpatient database in Japan that contains administrative claims and discharge data, which are collected over 6 months (from July 1st to December 31st) each year. All 82 university teaching hospitals are obliged to adopt the DPC system, but adoption by community hospitals is voluntary. Approximately 3.5 million inpatients’ discharge data were collected between July 1st and December 31st in 2010, accounting for about 45% of all acute care inpatient admissions in Japan.

The database includes the following information: patient age and sex; diagnoses, comorbidities at admission, and complications after admission recorded with the International Classification of Disease and Related Health Problems, 10th edition (ICD-10) codes; discharge status; referral status (presence of referral from outside clinics or hospitals for hospitalization); admitting hospital’s academic status (whether or not it is a university teaching hospital). There are three types of hospitalization diagnoses recorded in the DPC database; “admission diagnosis”, “main diagnosis” and “the diagnosis for which the largest resource was used for treatment during that hospitalization”. We used the last diagnosis as our principal diagnosis to select the patients from the database, following the common practice in studies conducted using DPC data. This seemed most appropariate because having the diagnoses codes for pneumonia in this diagnosis category was what triggered the pneumonia severity to be recorded into the database. Since 2010, DPC has recorded pneumonia severity indices for all patients discharged with principal diagnosis (as defined above) of pneumonia. Based on the recommendations from the Japanese Respiratory Society [25], data on the following five binary indices, similar to CURB65 score indices developed by the British Thoracic Society [4], were collected: (1) patient age ≥75 years old for females or ≥70 years old for males; (2) Blood Urea Nitrogen (BUN) ≥21 mg/dL or signs of dehydration present; (3) SpO_2_ ≤ 90% (or PaO_2_ ≤ 60 Torr); (4) presence of cognitive disturbance caused by pneumonia measured by the Japan Coma Scale [26] (which is well correlated with the Glasgow Coma Scale [27]); and (5) Systolic Blood Pressure (SBP) ≤90 mmHg. We utilized the latter four severity indices for this study, since all our patients were less than 65 years old.

This study was based on a secondary analysis of the administrative claims data. Given the anonymous nature of the data, the requirement for informed consent was waived. Study approval was obtained from the Institutional Review Board of University of Tokyo (Tokyo, Japan).

### Study patients

We identified patients aged 18–64 years in the DPC database in 2010 who were discharged from the hospitals with a principal diagnosis of pneumonia. We excluded those with a diagnosis of aspiration pneumonia and with diagnoses pertaining to animmunocompromised host. Specifically, hospitalizations with the following ICD-10 diagnostic codes were included: J10 (influenza due to identified influenza virus), J11 (influenza, virus not identified), J12 (viral pneumonia, not elsewhere classified), J13 (pneumonia due to *Streptococcus* pneumoniae), J14 (pneumonia due to *Haemophilus influenzae*), J15 (bacterial pneumonia, not elsewhere classified, except for J15.1: pneumonia due to *Pseudomonas*), J16 (pneumonia due to other infectious organisms, not elsewhere classified), J17 (pneumonia in diseases classified elsewhere, except for J17.2: pneumonia in mycoses), J18 (pneumonia, organism unspecified, except for J18.2: hypostatic pneumonia, unspecified), and A48.1 (Legionnaires’ disease).

### Hospital case volume

Hospital case volume was defined as the number of pneumonia patients discharged from each hospital during the 6-month period of the study. We examined the distribution of the hospitals with different case volumes, and categorized the hospitals into four groups (very low-, low-, medium-, and high-volume) based on the total case volume quartiles.

### Study outcome

The end point considered in the study was in-hospital death.

### Covariates

Patient age, sex, and comorbidities were extracted from the database. Comorbidities were converted into Charlson Comorbidity Index (CCI) scores based on Quan’s protocol [28]. The information on the severity of pneumonia at admission was also collected using the codes described above. Information on referral from other hospitals or clinics, and the teaching status of the hospital were also collected from the database.

For subgroup analysis, the four binary indices of pneumonia severity as described above were summed to calculate a modified A-DROP score (0 to 4) for each patient. The original A-DROP score has been shown to predict 30-day mortality of community acquired pneumonia patients similar to CURB-65 [29].

### Statistical analysis

We performed univariate comparisons of patient characteristics and in-hospital mortality among the four hospital case volume groups using the chi-squared test. Patient age was categorized into three groups: 18–44, 45–54, and 55–64 years old. The CCI was categorized into four levels: 0, 1–2, 3–6, and ≥7. We drew a histogram of the case volume across all of the hospitals, and described the variability of the hospital specific mortality among all hospitals and by volume level, by reporting the range of mortality and percentage of hospitals with zero in-hospital deaths.

Logistic regression analyses were performed to determine the association between hospital case volume and in-hospital mortality, adjusting for patient characteristics and type of hospital (academic hospital or not). We constructed two models for the adjusted analysis: model 1 with adjustment for hospital academic status and patient demographics and comorbidity index, but excluding pneumonia severity indices (i.e., age, sex, CCI, and referral status): model 2 with further adjustment by the inclusion of pneumonia severity indices. To account for potential clustering at the hospital level, we used Generalized Estimating Equations (GEEs) with a pre-specified exchangeable correlation structure for analysis [30], using the PROC GENMOD procedure with repeated coding of hospital identifiers.

For subgroup analysis, patients were stratified into five groups of pneumonia severity according to modified A-DROP scores. In-hospital mortality was calculated for each hospital case volume level in each of the five groups, and a test for trend was performed using Mantel-Haenszel Chi-squared statistic. For the groups with a statistically significant test result, we performed logistic regression analysis to determine the association between case volume and in-hospital mortality after adjusting for patient age, sex, and CCI. Because of the small sample size in the subgroup analysis, we included both age and CCI as continuous variables in the model to maintain the event per variable ratio above 10 [31]. All statistical tests were two-tailed, and factors were considered statistically significant at an α level of 0.05. All statistical analyses were performed using SAS version 9.2 (SAS Institute Inc., Cary, NC, USA).

## Results

### Study patients

**Figure 1:**
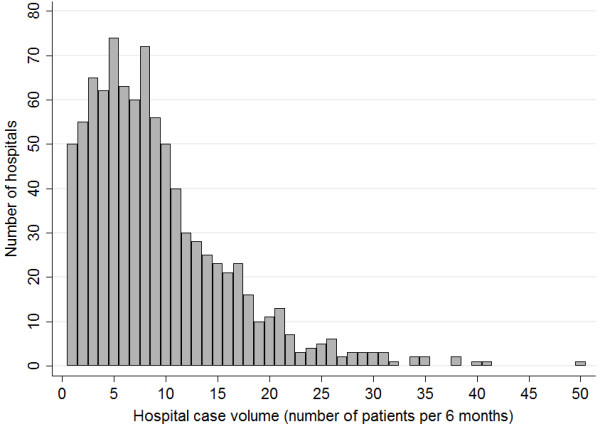
Distribution of case volume of non-elderly pneumonia patients over 6 months among hospitals enrolled in the DPC database 2010.

We identified a total of 10,936 cases of pneumonia hospitalizations among the non-elderly population during the study period. After excluding cases which did not meet the inclusion criteria, the final study population consisted of 8,293 cases of pneumonia at 896 hospitals. The number of hospitals and the different case volumes are shown in Figure 1. The number of cases of non-elderly pneumonia at each hospital was quite limited, with the majority of the hospitals treating less than 10 cases during the 6-month study period. Based on the total case volume quartiles, the hospitals were categorized into very low (1–7 patients per 6 months), low (8–11 patients per 6 months), medium (12–17 patients per 6 months), and high (18–50 patients per 6 months) volume groups.

In total, 273 in-hospital deaths (3.3%) were recorded (Table 1). The lower case volume groups had a slightly higher proportion of older patients and patients with higher CCI scores, although not statistically significant. Two of the pneumonia severity indices (BUN ≥ 21 mg/dL or signs of dehydration, and SBP ≤ 90 mmHg) were more prevalent in the lowest case volume group, while the proportions of the other two pneumonia severity indices (SpO_2_ ≤ 90% or PaO_2_ ≤ 60 Torr and presence of cognitive disturbance resulting from pneumonia) were quite similar among the four groups. When examining in-hospital mortality at the hospital level, the majority of hospitals (75% overall) had no in-hospital deaths among the non-elderly patients, and as expected, the proportion of hospitals with 0% mortality decreased from 86 to 53% as the case volume level increased (Table 2).

**Table 1:** Patient characteristics and in-hospital mortality overall and by hospital case volume level for discharges with a principal diagnosis of pneumonia, Japanese DPC database, 2010

	**Total**	**Very low (≤7)**	**Low (8–11)**	**Medium (12–17)**	**High (≥18)**	**p**^**†**^
Number of patients	8293	1771	2020	2146	2356	
Sex (male, %)	57.6%	59.7%	59.3%	55.5%	56.4%	0.01
Age						0.11
18–44 years	37.9%	36.8%	36.5%	40.5%	37.4%	
45–54 years	15.7%	15.3%	15.6%	15.5%	16.3%	
55–64 years	46.4%	47.9%	47.8%	44.0%	46.3%	
Charlson comorbidity index						0.16
0	43.9%	44.0%	42.2%	43.1%	46.1%	
1 or 2	42.8%	42.3%	43.3%	43.2%	42.2%	
3–6	11.1%	11.2%	12.0%	11.4%	10.0%	
7 and above	2.2%	2.6%	2.4%	2.2%	1.7%	
Referred from clinics/other hospitals	36.9%	36.0%	35.6%	42.4%	33.5%	<.001
Severity Indices						
BUN ≥21 mg/dL or signs of dehydration	19.8%	22.7%	19.7%	19.3%	18.4%	0.01
SpO_2_ ≤ 90% (or ≤ PaO_2_ 60 Torr)	22.1%	21.0%	22.7%	21.9%	22.5%	0.60
Presence of cognitive disturbance	8.0%	8.0%	8.0%	8.4%	7.6%	0.79
Systolic blood pressure ≤90 mmHg	7.6%	9.3%	8.2%	7.1%	6.4%	0.004
Academic hospital	12.1%	11.5%	14.9%	11.1%	10.9%	<.001
In-hospital death	3.3%	3.8%	3.6%	3.4%	2.6%	0.14

^†^p-value from chi-squared test for independence.

BUN: blood urea nitrogen; SpO_2_: oxygen saturation; PaO_2_: arterial oxygen tension.

**Table 2:** Hospital level mortality of non-elderly patients discharged with a principal diagnosis of pneumonia overall and by hospital case volume level, Japanese DPC database, 2010

	**Total**	**Very low (≤7)**	**Low (8–11)**	**Medium (12–17)**	**High (≥18)**
Number of patients	8293	1771	2020	2146	2356
Number of hospitals	896	429	218	150	99
% of hospitals with zero mortality	75	86	72	65	53
% range of mortality rates	0-100	0-100	0-27	0-23	0-16

### Volume-outcome relationship in the overall population

In the unadjusted logistic regression analysis in the overall population, an inverse trend for the risk of death with hospital case volume was observed (Table 3). However, after adjusting for age, sex, CCI, presence of referral from other clinics or hospital, and hospital academic status, the odds ratios (ORs) of death in the three lower case volume groups compared with the highest case volume groups became statistically insignificant with point estimates very close to 1.0 (Table 3; adjusted model1). Further adjustment for pneumonia severity indices, did not substantially change the estimated ORs (Table 3; adjusted model2). In this final model, all four severity indices were significantly associated with the risk of in-hospital death, with the indicator for cognitive impairment having the largest OR of 5.85 (95% confidence interval (CI): 4.18–8.18).

**Table 3:** Unadjusted and adjusted logistic regression models for the risk of in-hospital mortality for patients with a principal diagnosis of pneumonia, Japanese DPC database, 2010

	**Unadjusted analysis**				**Adjusted model 1**				**Adjusted model 2**			
	**OR**	**95% CI**		**p**	**OR**	**95% CI**		**p**	**OR**	**95% CI**		**p**
Hospital case volume (vs. high)												
Very low	1.48	1.04	2.10	0.03	1.12	0.90	1.38	0.32	1.02	0.79	1.31	0.88
Low	1.39	0.98	1.96	0.06	1.03	0.83	1.27	0.80	1.13	0.88	1.46	0.34
Medium	1.32	0.92	1.90	0.13	1.06	0.85	1.33	0.59	1.03	0.80	1.32	0.83
Age (year) (vs. 55–64)												
18–44					0.43	0.32	0.58	<.001	0.48	0.35	0.67	<.001
45–54					1.24	0.96	1.61	0.10	1.30	0.98	1.73	0.07
Charlson comorbidity index (vs. 0)												
1 or 2					1.46	1.05	2.04	0.03	1.35	0.93	1.97	0.11
3–6					3.00	2.03	4.43	<.001	2.59	1.63	4.09	<.001
7 and above					10.37	6.49	16.57	<.001	11.04	6.25	19.49	<.001
Female (vs. male)					1.63	1.24	2.15	<.001	1.38	1.02	1.87	0.04
Referred from clinics/other hospitals (vs. not)					0.97	0.75	1.25	0.80	1.20	0.88	1.63	0.61
Academic hospital (vs. non-academic)					1.13	0.83	1.56	0.44	1.06	0.86	1.31	0.58
Severity indices												
BUN ≥21 mg/dL or signs of dehydration									2.44	1.80	3.31	<.001
SpO_2_ ≤ 90% (or ≤ PaO_2_ 60 Torr)									2.26	1.61	3.16	<.001
Presence of cognitive disturbance									5.85	4.18	8.18	<.001
Systolic blood pressure ≤90 mmHg									2.83	1.97	4.07	<.001

OR: odds ratio; CI: confidence interval.

BUN: blood urea nitrogen; SpO_2_: oxygen saturation; PaO_2_: arterial oxygen tension.

### Volume outcome relationship stratified by pneumonia severity

When examining the relationship between hospital case volume and in-hospital mortality stratified by A-DROP scores, we observed a strong correlation between A-DROP score and in-hospital mortality rate (Table 4). Among the five severity strata, a statistically significant inverse trend for mortality against increasing hospital case volume was observed only at the level of A-DROP score 2. The results of the regression analysis for this group are shown in Table 5. After adjusting for age, CCI, and sex, the OR of in-hospital death for very low vs. high hospital case volume was statistically significant at 2.70 (95% CI 1.12–6.55) and for low vs. high hospital case volume was borderline insignificant at 2.40 (95% CI 0.99–5.83).

**Table 4:** In-hospital mortality for pneumonia patients by hospital case volume level, after stratification by A-DROP score calculated from the Pneumonia Severity Indices at admission, Japanese DPC database, 2010

**A-DROPScore**			**Hospital case volume level**				
		Total^*^	**Very low (≤7)**	**Low (8–11)**	**Medium (12–17)**	**High (≥18)**	**p**^**†**^
0	N	4906	1027	1193	1283	1403	0.31
	Deaths	29	9	6	7	7	
	%	0.6	0.9	0.5	0.6	0.5	
1	N	1981	421	487	524	549	0.72
	Deaths	61	6	22	20	13	
	%	3.1	1.4	4.5	3.8	2.4	
2	N	701	158	184	176	183	0.01
	Deaths	58	18	20	12	8	
	%	8.3	11.4	10.9	6.8	4.4	
3	N	220	48	43	61	68	0.76
	Deaths	52	14	7	15	16	
	%	23.6	29.2	16.3	24.6	23.5	
4	N	120	34	34	28	24	0.36
	Deaths	53	14	14	12	13	
	%	44.2	41.2	41.2	42.9	54.2	

^†^p-value from Mantel-Haenszel chi-squared test for trend.

*365 patients had missing value in at least one of the severity markers and were excluded from the table.

**Table 5:** Unadjusted and adjusted logistic regression models for the risk of in-hospital death associated with community acquired pneumonia, Japanese DPC database, 2010

	**Unadjusted analysis**				Adjusted model*			
	**OR**	**95% CI**		**p**	**OR**	**95% CI**		**p**
Hospital case volume (vs. high)								
Very low	2.69	1.13	6.40	0.03	2.70	1.12	6.55	0.03
Low	2.51	1.06	5.95	0.04	2.40	0.99	5.83	0.05
Medium	1.51	0.60	3.81	0.38	1.31	0.49	3.49	0.59
Age (year)					1.02	1.00	1.05	0.10
Female (vs. male)					0.66	0.37	1.20	0.18
CCI					1.30	1.14	1.48	<.001

*Adjusted for age, sex and CCI (Age and CCI as continuous variables).

CCI: Charlson Comorbidity Index; OR: odds ratio; CI: confidence interval.

## Discussion

Contrary to our initial hypothesis, we found no statistically significant association between hospital case volume and in-hospital mortality in the overall population of non-elderly pneumonia patients, after adjustments for patient demographics, comorbidities, severity of pneumonia, presence of referral to the hospital, and hospital teaching status. After stratifying the population by A-DROP pneumonia severity score, a larger casevolume was found to be associated with lower in-hospital mortality in the subgroup with intermediate severity (A-DROP score = 2). No trend was observed in the other subgroups with mild severity (A-DROP score = 0 or 1) or high severity (A-DROP score = 3 or 4).

Most of the previous studies in pneumonia patients have been limited to the elderly population. To the best of our knowledge, this is the first study that explicitly examined the volume-outcome relationship of pneumonia patients in the non-elderly population; a population for which we considered that the relationship could not readily generalized from that in the elderly pneumonia population.

Previous published studies on the volume-outcome relationship in pneumonia patients have shown mixed results. In a Medicare patient population, Lindenauer and colleagues showed that while in-hospital mortality and pneumonia case volume were inversely associated, the risk of 30-day mortality among different hospital volume groups were similar [17]. However, Ross and colleagues reported a statistically significant association between log-transformed volume and in-hospital mortality [16]. Marrie and colleagues investigated an adult population in Alberta, Canada, and found an insignificant contribution by hospital volume for prediction of in-hospital death [18]. These inconsistencies could result from differences in populations, methods, level of case-mix adjustment, and statistical modelling. However, it could also have arisen from modification of the association by disease severity.

Our results indicate a possible modification of hospital volume-outcome relationship by pneumonia severity. Few previous studies focused their analysis on identifying the effect of disease severity on the relationship between process of care measures and outcome in medically treated patients. Shahin and colleagues studied the association between hospital case volume and in-hospital death in sepsis patients [32], and Houck and colleagues studied the association between outcome and timely administration of antibiotics in elderly pneumonia patients [24], both of which investigated the association stratified by disease severity. They reported no substantial effect of severity on the association. On the other hand, Lin and colleagues [22], in their study on the relationship between physician case volume and in-hospital mortality of pneumonia patients treated in Intensive Care Units in Taiwan, reported an attenuated strength of association in the severest (acute respiratory failure) patient group. Also, Durairaj and colleagues reported an association between hospital case volume and outcome among intensive care patients only in the group with more severe disease [23]. Evidence is still lacking and there are mixed results, but our results together with those of Lin and colleagues and Durairaj and colleagues suggest a need for stratification by disease severity when evaluating the relationship between process of care and outcome. Proper assessment of factors modifying the association will not only reveal the true relationship between case volume and outcome, but will also allow identification of groups that could most successfully benefit from intervention.

In our study, the hospital volume-outcome relationship was only evident in the intermediate pneumonia severity level. A possible clinical explanation for this unique pattern is that in non-elderly patients, very severe pneumonia cases are mostly not very responsive to the level of variations in the treatments reflected in hospital case volume, with outcome depending much more on the patient’s baseline status, including comorbidities and pneumonia severity. This is consistent with the report from Marrie and colleagues, where deaths resulting from community-acquired pneumonia among young and middle aged patients were more related to the severity of disease and less to the organisation of care management [33]. It is also possible that the outcomes of cases of mild severity are not greatly compromised by the possibly lower quality of care at lower case volume hospitals. Thus, the impact of clinical management would be most reflected on those who were at “intermediate” severity.

The main strengths of the present study are three-fold. First, the study included a large number of non-elderly pneumonia cases using a nationwide database. Second, we focused on evaluating the specific effect of hospital case volume on the outcomes of pneumonia treatment explicitly in a non-elderly population. Third, because of incorporation of pneumonia severity indices in the DPC database, we were able to both extensively adjust for the case mix in the overall analysis and conduct subgroup analyses within well defined severity levels.

There are some limitations to our study which should be noted. First, the study was based on administrative data which are less well validated compared with well-designed prospective cohorts or registries. Unfortunately, no validation study of pneumonia diagnoses has been conducted in the DPC database, which may cause potential misclassification. We defined the diagnosis such that all included patients had information on the pneumonia severity index, minimizing the possibility of non-pneumonia cases being included in the cohort. Second, there were some missing data for patient level covariates. However, since this was less than 5% (380/8493), we do not believe that it would have grossly biased our estimates from our complete case analysis. Third, influential comorbid conditions might not have been completely captured in the CCI, and could have biased our results. Adjustments or stratifications by the severity indices should have alleviated this to a certain extent because of the correspondence between the two, but residual confounding is still possible. Fourth, we lacked information on pre-hospitalization treatment. Although we accounted for the presence of referrals from other clinics or hospitals, no information on pre-arrival treatment was collected, and thus could bias our estimates if different pre-arrival treatment patterns were present among the different case volume groups. Fifth, no information on hospital characteristics, other than academic status, was available for this study. It is possible that factors such as provider care ratios (bed-physician or bed-nurse ratio), tertiary care center status, urban/rural locale, or the presence of infection specialists or infection teams at the hospitals may explain or enhance some of the differences observed between the smaller case volume hospitals and larger case volume hospitals. Finally, because participation of community hospitals in the DPC database was voluntary, the population identified might not be completely representative of the whole population in Japan. Very small hospitals are known to have a low participation rate in the DPC system, and thus the overall mortality may be underestimated by this. Also, since the DPC database captures hospitalization that occurred only between July and December, we are not fully capturing the seasonal occurrence of pneumonia. Lin and colleagues previously reported a seasonal trend of pneumonia admissions that starts in November and peaked through March in all adult age groups [34]. If the seasonality of pneumonia occurs differentially among the hospitals, misclassification of the case volume level may bias our results.

## Conclusions

Larger hospital case volume was associated with lower in-hospital mortality in non-elderly patients with pneumonia of intermediate severity. No significant volume-outcome relationships were observed for the patients with mild or high severity pneumonia. This result supports future interventions for quality of care improvements to be targeted towards hospitals with lower case volume to improve treatment outcomes particularly in patients admitted with intermediate pneumonia severity.

## Competing interests

The authors declare they have no competing interests.

## Authors’ contributions

HK designed the study, analyzed and interpreted data, and prepared manuscript. YT supported the analyses of data and the drafting of manuscript. HY and HHo were involved in acquisition of data, and supported the analyses/interpretation of data, and drafted the manuscript. KKK took part in interpretation of data and manuscript preparation. HHa was involved in the design of study, interpretation of data, and manuscript preparation. All authors read and approved the final manuscript.

## Pre-publication history

The pre-publication history for this paper can be accessed here:

http://www.biomedcentral.com/1472-6963/14/302/prepub
